# Supplementary material for: Heparin-based hydrogel scaffolding alters the transcriptomic profile and increases the chemoresistance of MDA-MB-231 triple-negative breast cancer cells
Source: Biomater Sci. 2020 Feb 13;8(10):2786–96. doi: 10.1039/c9bm01481k (PMC7497406; doi:10.1039/c9bm01481k)
Supplement: Supplementary file 2 [file BM-008-C9BM01481K-s002.zip › Supplementary File 4/GSEA.top50.pdf]

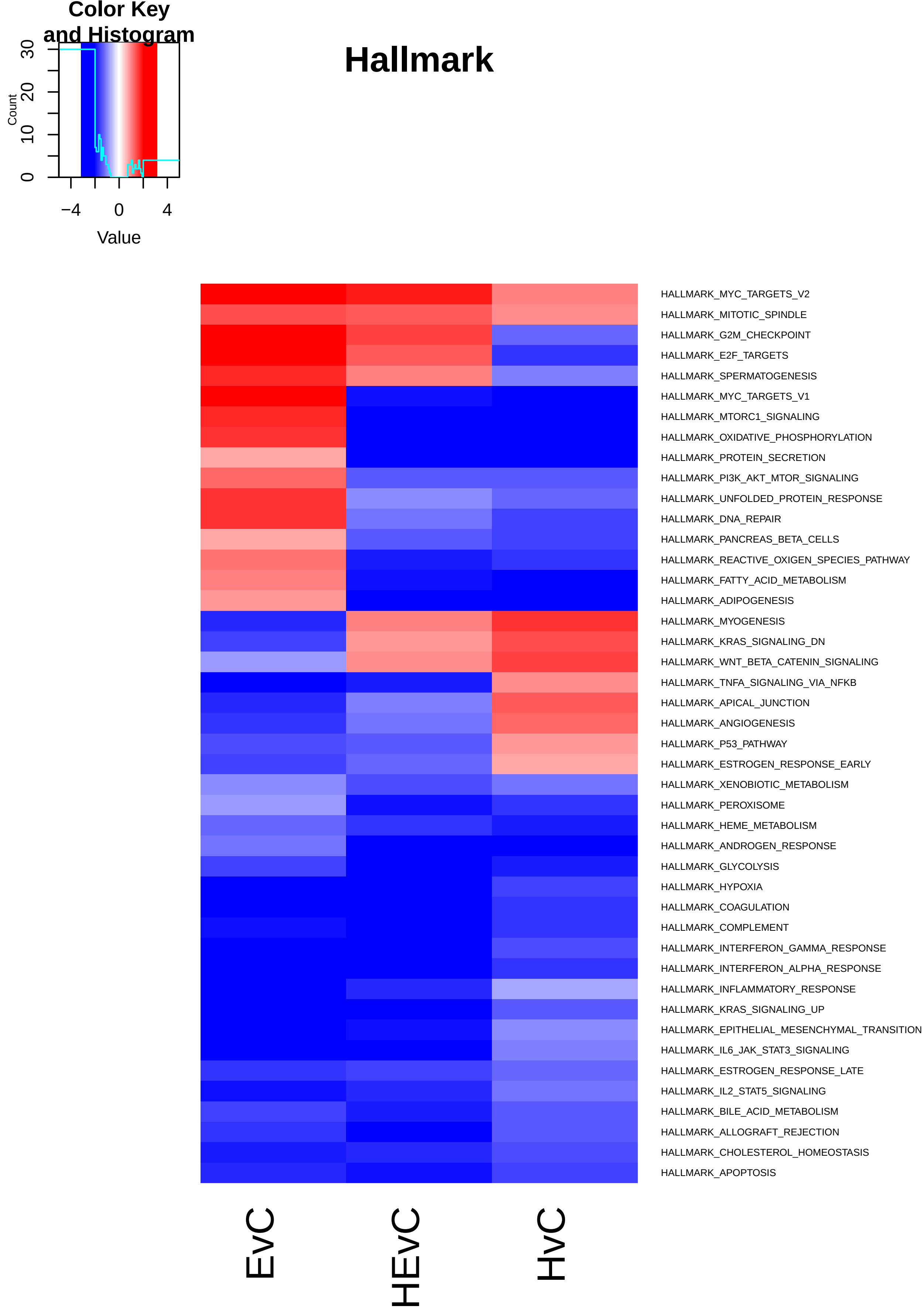
Color Key
and Histogram
Count
0
10
20
30
−4
0
4
Value
Hallmark
HALLMARK_MYC_TARGETS_V2
HALLMARK_MITOTIC_SPINDLE
HALLMARK_G2M_CHECKPOINT
HALLMARK_E2F_TARGETS
HALLMARK_SPERMATOGENESIS
HALLMARK_MYC_TARGETS_V1
HALLMARK_MTORC1_SIGNALING
HALLMARK_OXIDATIVE_PHOSPHORYLATION
HALLMARK_PROTEIN_SECRETION
HALLMARK_PI3K_AKT_MTOR_SIGNALING
HALLMARK_UNFOLDED_PROTEIN_RESPONSE
HALLMARK_DNA_REPAIR
HALLMARK_PANCREAS_BETA_CELLS
HALLMARK_REACTIVE_OXIGEN_SPECIES_PATHWAY
HALLMARK_FATTY_ACID_METABOLISM
HALLMARK_ADIPOGENESIS
HALLMARK_MYOGENESIS
HALLMARK_KRAS_SIGNALING_DN
HALLMARK_WNT_BETA_CATENIN_SIGNALING
HALLMARK_TNFA_SIGNALING_VIA_NFKB
HALLMARK_APICAL_JUNCTION
HALLMARK_ANGIOGENESIS
HALLMARK_P53_PATHWAY
HALLMARK_ESTROGEN_RESPONSE_EARLY
HALLMARK_XENOBIOTIC_METABOLISM
HALLMARK_PEROXISOME
HALLMARK_HEME_METABOLISM
HALLMARK_ANDROGEN_RESPONSE
HALLMARK_GLYCOLYSIS
HALLMARK_HYPOXIA
HALLMARK_COAGULATION
HALLMARK_COMPLEMENT
HALLMARK_INTERFERON_GAMMA_RESPONSE
HALLMARK_INTERFERON_ALPHA_RESPONSE
HALLMARK_INFLAMMATORY_RESPONSE
HALLMARK_KRAS_SIGNALING_UP
HALLMARK_EPITHELIAL_MESENCHYMAL_TRANSITION
HALLMARK_IL6_JAK_STAT3_SIGNALING
HALLMARK_ESTROGEN_RESPONSE_LATE
HALLMARK_IL2_STAT5_SIGNALING
HALLMARK_BILE_ACID_METABOLISM
HALLMARK_ALLOGRAFT_REJECTION
HALLMARK_CHOLESTEROL_HOMEOSTASIS
HALLMARK_APOPTOSIS
EvC
HEvC
HvC
